# Super Enhancer Profiles Identify Key Cell Identity Genes During Differentiation From Embryonic Stem Cells to Trophoblast Stem Cells Super Enhencers in Trophoblast Differentiation

**DOI:** 10.3389/fgene.2021.762529

**Published:** 2021-10-12

**Authors:** Rongpu Jia, Yu Gao, Song Guo, Si Li, Liangji Zhou, Chenyu Gou, Yijuan Huang, Meiqiong Fan, Yuanqiu Chen

**Affiliations:** The Sixth Affiliated Hospital of Sun Yat-Sen University, Guangzhou, China

**Keywords:** trophoblast stem cell, embryonic stem cell, super enhancer, H3K27ac, H3K4me1

## Abstract

Trophoblast stem cells (TSCs) are derived from blastocysts and the extra-embryonic ectoderm (ExE) of post-implantation embryos and play a significant role in fetal development, but the roles that TSCs play in the earlier status of fetal diseases need further exploration. Super enhancers (SEs) are dense clusters of stitched enhancers that control cell identity determination and disease development and may participate in TSC differentiation. We identified key cell identity genes regulated by TSC-SEs via integrated analysis of H3K27ac and H3K4me1 chromatin immunoprecipitation sequencing (ChIP-seq), RNA-sequencing (RNA-seq) and ATAC-sequencing (ATAC-seq) data. The identified key TSC identity genes regulated by SEs, such as epidermal growth factor receptor (EGFR), integrin β5 (ITGB5) and Paxillin (Pxn), were significantly upregulated during TSC differentiation, and the transcription network mediated by TSC-SEs enriched in terms like focal adhesion and actin cytoskeleton regulation related to differentiation of TSCs. Additionally, the increased chromatin accessibility of the key cell identity genes verified by ATAC-seq further demonstrated the regulatory effect of TSC-SEs on TSC lineage commitment. Our results illustrated the significant roles of the TSC-SE-regulated network in TSC differentiation, and identified key TSC identity genes EGFR, ITGB5 and Pxn, providing novel insight into TSC differentiation and lays the foundation for future studies on embryo implantation and related diseases.

## Introduction

Trophoblasts play a significant role in embryonic development, and many studies have demonstrated that developmental disorders of trophoblasts are closely related to fetal diseases (Knöfler et al., 2019). Nevertheless, few studies have proven the role of abnormal trophoblast stem cells in fetal development. Understanding the key regulatory network involved in the processes of trophoblast stem cell (TSC) differentiation and gene expression is important for the further study of TSC differentiation, embryo implantation and embryonic development.

After 3–4 days of fertilization, cells differentiate into the trophectoderm and inner cell mass (ICM), after which blastocysts formed. The ICM has the potential to form an embryo, and the trophectoderm will develop into extraembryonic structures such as trophoblast stem cell and placenta (Pijnenborg et al., 1981; Gamage et al., 2016; Turco et al., 2018). The isolation of the trophectoderm and ICM is crucial for embryonic development and plays an important role in embryo implantation. Implantation failure could lead to severe pregnancy-related outcomes, such as abortion and preeclampsia (Wu et al., 2008). Trophoblast lineages are considered developed from TSCs which also are crucial in fetal diseases. However, it is difficult to separate and cultivate TSC *in vitro*. Embryonic stem cells (ESCs) are derived from the ICM, possess multipotential differentiation potency, and can be acquired through separation and cultivation *in vitro* (Thomson et al., 1998); which make them an excellent model for research on TSC differentiation *in vitro*.

Super enhancers (SEs) are defined as clusters of enhancers consisting of abundant transcription factor binding and histone marks, such as the monomethylation of lysine 4 of histone H3 (H3K4me1) and the acetylation of lysine 27 of histone H3 (H3K27ac), which are closely related to enhancer activity and the expression of the closest proximal gene (Pott and Lieb, 2015). SEs were first identified in ESCs and shown to be strongly bound by master transcription factors (Thomson et al., 1998). Previous studies have shown that SEs can affect the process of differentiation from mononuclear cytotrophoblasts (CytT) to multinuclear syncytiotrophoblasts (SynT) (Kwak et al., 2019), but it remains unknown what roles SEs play in the process of TSC differentiation.

In this study, we identified the crucial cell identity genes regulating TSC differentiation *via* an integrated analysis of chromatin immunoprecipitation sequencing (ChIP-seq) and RNA sequencing (RNA-seq) data, and we constructed a comprehensive network containing 228 upregulated genes mediated by TSC-unique SEs. According to the results of this study, we have discovered new potential targets for TSC differentiation, and we provide evidence for future research on ESC differentiation, thereby laying the foundation for studying the mechanisms of embryo implantation and related diseases.

## Methods

### Data Sources

B6TS H3K27ac and H3K4me1 ChIP-seq data from GSE109250, TSC H3K27ac and H3K4me1 ChIP-seq data, TSC and ESC ATAC-seq data in Supplementary Figure S1 and RNA-seq data of TSCs and ESCs from GSE110950, and ATAC-seq data of TSCs and ESCs from GSE94694 were downloaded from the GEO database.

### Chromatin Immunoprecipitation-Sequencing Data Analysis

Raw reads were filtered and trimmed to remove sequencing adaptors and low-quality reads using TrimGalore (v0.6.0). After the application of quality control procedures, the clean reads were mapped to the *Mus musculus* genome (version mm10) using Bowtie2 (v2.5.1). The unique mapped reads were preserved, and others were eliminated. Peaks were called using MACS2 (v.2.1.1) with the parameters -m 5 50 -p 1e-5 -g hs for H3K27ac and H3K4me1 ChIP-seq data and --nomodel --shift -75 --extsize 150 -g hs for ATAC-seq data. Reads were normalized [in reads per kilobase per million (RPKM)], and heatmaps were drawn using deepTools (v2.3.6.0). The visualization of normalized ChIP-seq data was performed using the Integrative Genomics Viewer (IGV).

### Identification of Super Enhancers

Enhancers were defined as peak regions of enhancer markers such as H3K27ac and H3K4me1 that were situated at least 2 kb from annotated transcription start sites (TSSs). Enhancer peaks within 12.5 kb were stitched together as an individual unit for further signal ranking to identify SEs using the ROSE algorithm (version 2). Each enhancer was ranked according to the signal of H3K27ac or H3K4me1 in the genomic region, including stitched enhancers and individual enhancers without neighboring enhancers within 12.5 kb. Stitched or individual enhancers with an H3K27ac or H3K4me1 signal exceeding the signal of the enhancer located at the point where the slope of the signal curve in the H3K27ac or H3K4me1 ChIP-seq intensity distribution plot was 1 were considered SEs. Enhancers with weaker signals were considered TEs. The annotation of TEs and SEs was performed with homer (v4.11.1) with the default parameters. The R package ChIPseeker (v1.20.0) was used to profile the distribution of SEs and TEs throughout the genome.

### RNA-Sequencing Data Processing

Sequencing adaptor-containing reads were removed by using SOAPnuke to filter the sequencing data, and clean reads were acquired and stored in FASTQ format. The clean reads were mapped to the mm10 reference genome for coverage calculation and quality control using HISAT2 (v2.0.4). The clean reads were aligned to the reference coding gene set by using Bowtie2 (v2.2.5), and the expression levels of the genes were then calculated with RSEM (v1.2.12). Pheatmap (v1.0.8) was used to draw a heatmap according to the gene expression profiles of different samples. DESeq2 (v1.4.5) was used to perform differential expression analysis with a thresholds of a fold change >2 and *q* value < 0.05.

### KEGG and GO Enrichment Analysis

The R package clusterProfiler (v3.11) was used to perform KEGG (https://www.kegg.jp/) and GO (http://www.geneontology.org/) enrichment analyses of annotated DEGs. The significance levels of terms and pathways were corrected according to the q value with a rigorous threshold (q value ≤ 0.05). The R package GOplot (v1.0.2) was used to generate the Circos plot of the GO enrichment analysis results.

### Gene Set Enrichment Analysis

To explore the intrinsic molecular mechanisms of TSC differentiation, GSEA was performed. To reveal all related terms, the expression matrix of RNA-seq data was used to perform the GSEA with all the terms in MSigDB. *p* < 0.05 was considered statistically significant.

### Establishment of the Protein-Protein Interaction Network

The SE-targeted upregulated genes were submitted to the STRING (v10.5; http://string‐db.org/) online database to construct the corresponding protein-protein interaction (PPI) network. The protein interaction matrix was exported and then imported to Cytoscape software (v3.6.1) to draw the interaction network to visualize and analyze the PPI network. The degrees of connectivity among genes were calculated with cytoHubba (v0.1).

## Results

### Identification of TSC-SEs via the Analysis of H3K27ac and H3K4me1 ChIP-Seq Data

**FIGURE 1:**
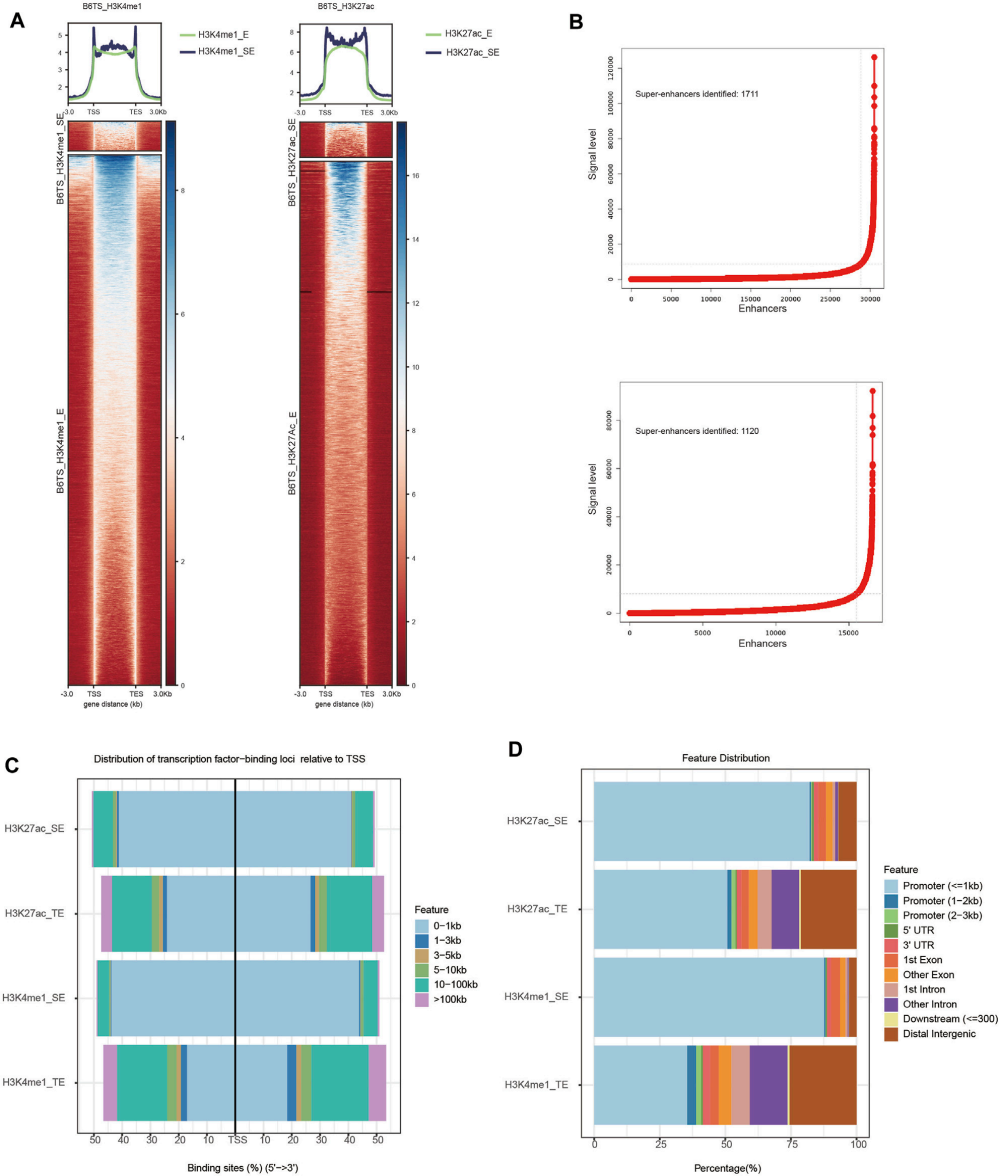
Profiles of SEs in TSCs (B6TS). **(A)** Heatmaps showing the average signal levels of H3K27ac and H3K4me1 SEs and TEs in TSCs (B6TS). **(B)** Identification of SEs. Enhancers within 12.5 kb were stitched together and ranked by the level of the H3K27ac and H3K4me1 signals. Enhancers with an H3K27ac signal above a cutoff of a slope of 1 on the curve were considered SEs. **(C)** Distribution of SEs and TEs throughout the genome (transcription factor-binding loci relative to TSS). **(D)** Distribution of SEs and TEs throughout the genome (distribution features).

To understand the roles of SEs in the differentiated TSCs, a ChIP-seq analysis of H3K27ac and H3K4me1 was performed to investigate whether SEs regulate the enhanced differentiation of TSCs. Heatmaps were plotted to illustrate the distribution of H3K27ac and H3K4me1 signal levels in differentiated TSCs (Figure 1A). SEs are composed of clusters of TEs and recruit many transcription factors and cofactors to promote transcription through high-density histone modifications. Thus, SEs play key roles in cell differentiation by regulating key cell identity genes. Enhancers within a 12.5 kb region marked by H3K27ac and H3K4me1 peaks were assembled and arranged according H3K27ac and H3K4me1 signal levels to identify SEs using the ROSE algorithm. The numbers of identified SEs marked by H3K27ac and H3K4me1 signals in TSCs were 1,120 and 1711, respectively (Figure 1B). The percentage of SEs positioned at a minimum distance from TSSs and promoters was approximately 1.5 times greater than that of TEs (Figure 1C). The distribution percentages and average signals of SEs and TEs marked by H3K27ac and H3K4me1 were identified. Little difference was observed between the groups marked by different signal labels. However, the overall signal intensity of SEs was higher than that of TEs in the same group (Figure 1D). In conclusion, SEs may play a more significant role than TEs in transcription because of their approximate spatial distance from TSSs and promoters.

### Differentially Expressed Genes Identification and Functional Enrichment Analysis

**FIGURE 2:**
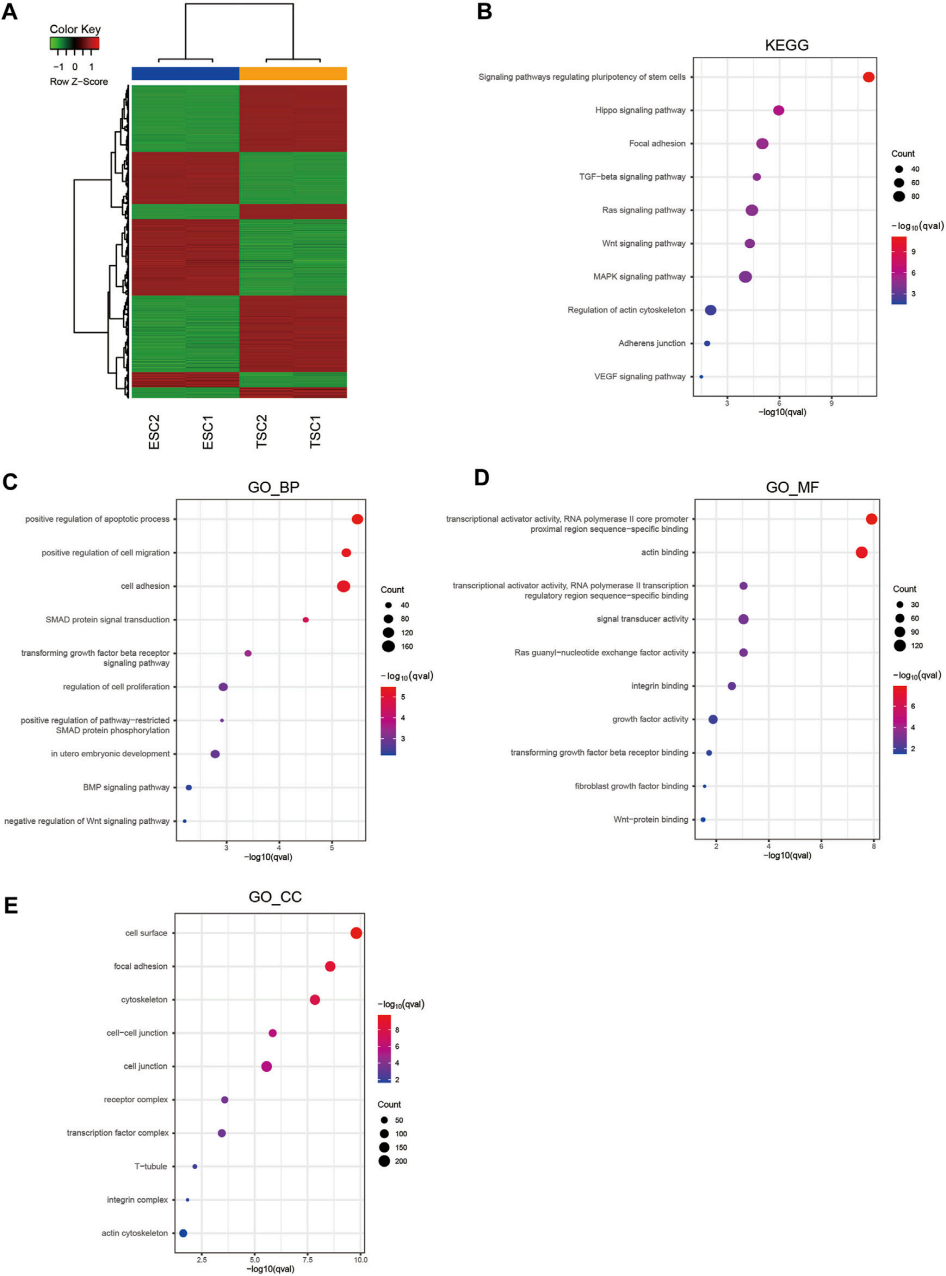
Hierarchical clustering and enrichment analysis of DEGs. **(A)** Heatmap showing the hierarchical clustering of DEGs. **(B)** KEGG pathway enrichment analysis of DEGs. **(C)** GO_BP enrichment analysis of DEGs. **(D)** GO_MF enrichment analysis of DEGs. **(E)** GO_CC enrichment analysis of DEGs.

To identify the key mechanism underlying TSC differentiation and the related molecular regulatory network, we compared gene expression between ESCs and TSCs using RNA-seq. It was evident in the heatmaps that there was a significantly distinct difference in the gene expression patterns of ESCs and TSCs, indicating that the gene expression network was markedly altered between these cell types (Figure 2A). Therefore, we intended to identify the key mechanisms mediating the differentiation of TSCs via functional enrichment analysis using the identified DEGs including the upregulated and downregulated genes. KEGG pathway analysis of DEGs showed enrichment in terms related to regulating the pluripotency of stem cells, signaling pathways regulating the pluripotency of stem cells, the Hippo signaling pathway and focal adhesion signa ling pathways (Figure 2B). The top 4 terms enriched in the GO biological process category (GO_BP) were positive regulation of apoptotic process, positive regulation of cell migration, cell adhesion and SMAD protein signal transduction signaling pathways (Figure 2C). In the GO molecular function category (GO_MF), functional enrichment analysis showed the enrichment of RNA polymerase II core promoter proximal region sequence-specific binding, RNA polymerase II transcription regulatory region sequence-specific binding actin binding, and signal transducer activity (Figure 2D). Moreover, in the GO cellular component category (GO_CC), functional enrichment analysis showed the enrichment of cell surface, focal adhesion and cytoskeleton signaling pathways (Figure 2E). In summary, functions related to adhesion, migration and the regulation of the actin cytoskeleton are active in differentiated TSCs.

### Integrative Analysis of DEGs and TSC-SEs

**FIGURE 3:**
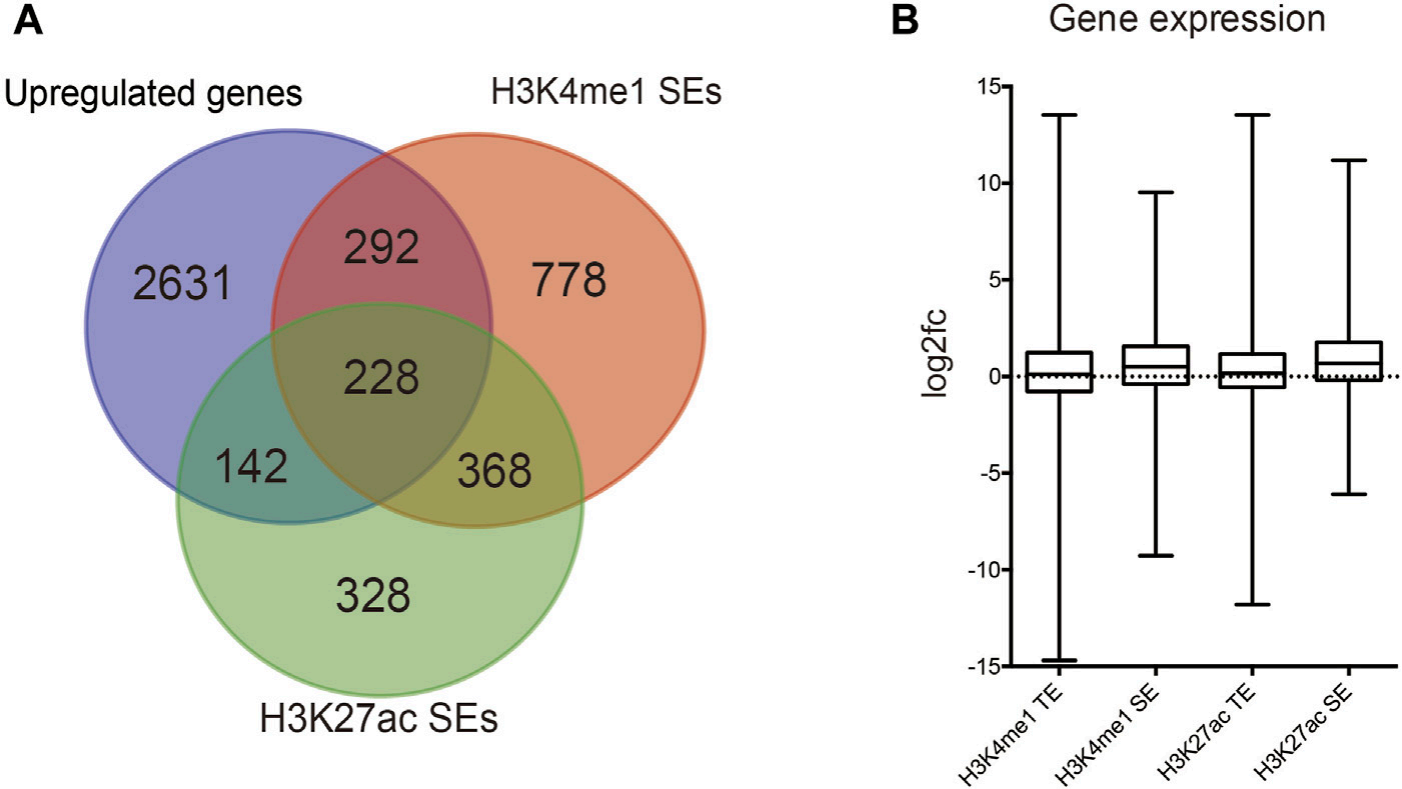
Identification of SE-regulated key cell identity genes. **(A)** Venn diagram showing the overlap between SEs marked by H3K27ac and H3K4me1 signals and upregulated genes in TSCs. **(B)** Relative gene transcript levels in SEs and TEs marked by H3K27ac and H3K4me1 signals.

**FIGURE 4:**
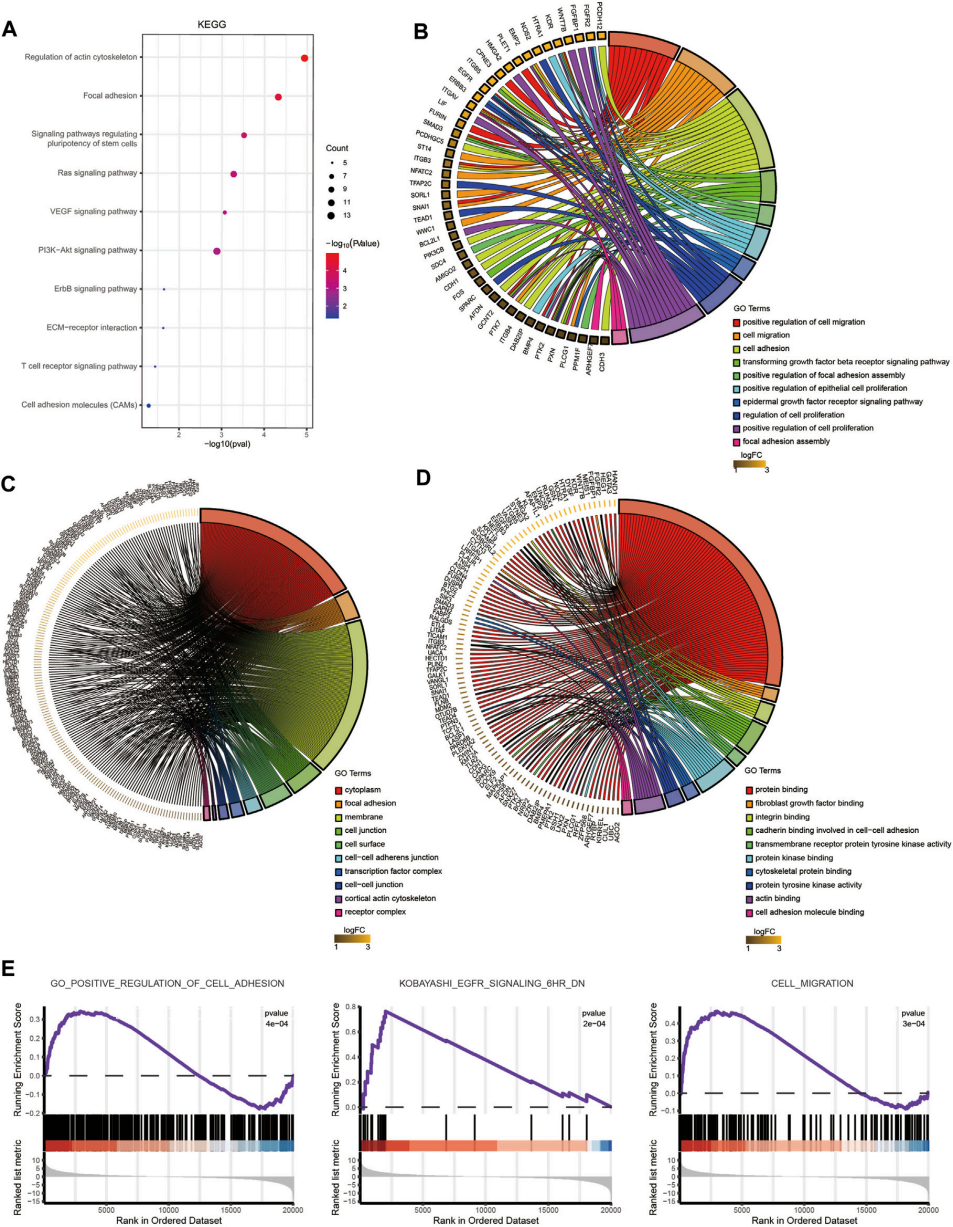
Enrichment analysis of TSC-SEs. **(A)** KEGG pathway enrichment analysis of upregulated genes targeted by TSC-SEs. **(B)** GO_BP pathway enrichment analysis of TSC-SEs. **(C)** GO_CC pathway enrichment analysis of TSC-SEs. **(D)** GO_MF pathway enrichment analysis of TSC-SEs. **(E)** Gene set enrichment analysis of TSC-SEs.

We identified DEGs and revealed the mechanisms of differentiation in TSCs through functional enrichment analysis. There was an urgent need to identify the core mechanism by reducing the scope. We obtained 228 distinct upregulated genes that could potentially be regulated by TSC-SEs by integrating the results of the H3K27ac, H3K4me1 and RNA-seq analyses (Figure 3A). Furthermore, we could see that the genes targeted by SEs presented higher fold-changes than the genes targeted by TEs (Figure 3B). This further proved that SEs have distinct effects on upregulating gene expression. The abovementioned 228 genes were analyzed by functional enrichment analysis to clarify the core gene expression network regulated by TSC-SEs. KEGG pathway analysis showed enrichment in the regulation of the actin cytoskeleton, focal adhesion and signaling pathways regulating the pluripotency of stem cells (Figure 4A). GO_BP functional enrichment analysis showed enrichment in the positive regulation of cell migration, cell adhesion, the positive regulation of cell proliferation and the cell migration signaling pathway (Figure 4B). GO_CC functional enrichment analysis showed enrichment in the cytoplasm and membrane signaling pathways (Figure 4C). GO_MF functional enrichment analysis showed enrichment in protein binding, protein kinase binding and cadherin binding involved in the cell-cell adhesion signaling pathway (Figure 4D). Furthermore, GSEA showed significant enrichment in cell adhesion, cell migration and the EGFR signaling pathway (Figure 4E). These results showed that the main signaling pathways were related to cell migration, adhesion and the regulation of the actin cytoskeleton. Therefore, SEs enhance gene expression at the level of transcription mainly through the signaling pathways mentioned above.

### Protein-Protein Interaction Network Construction and Key Cell Identity Gene Identification

**FIGURE 5:**
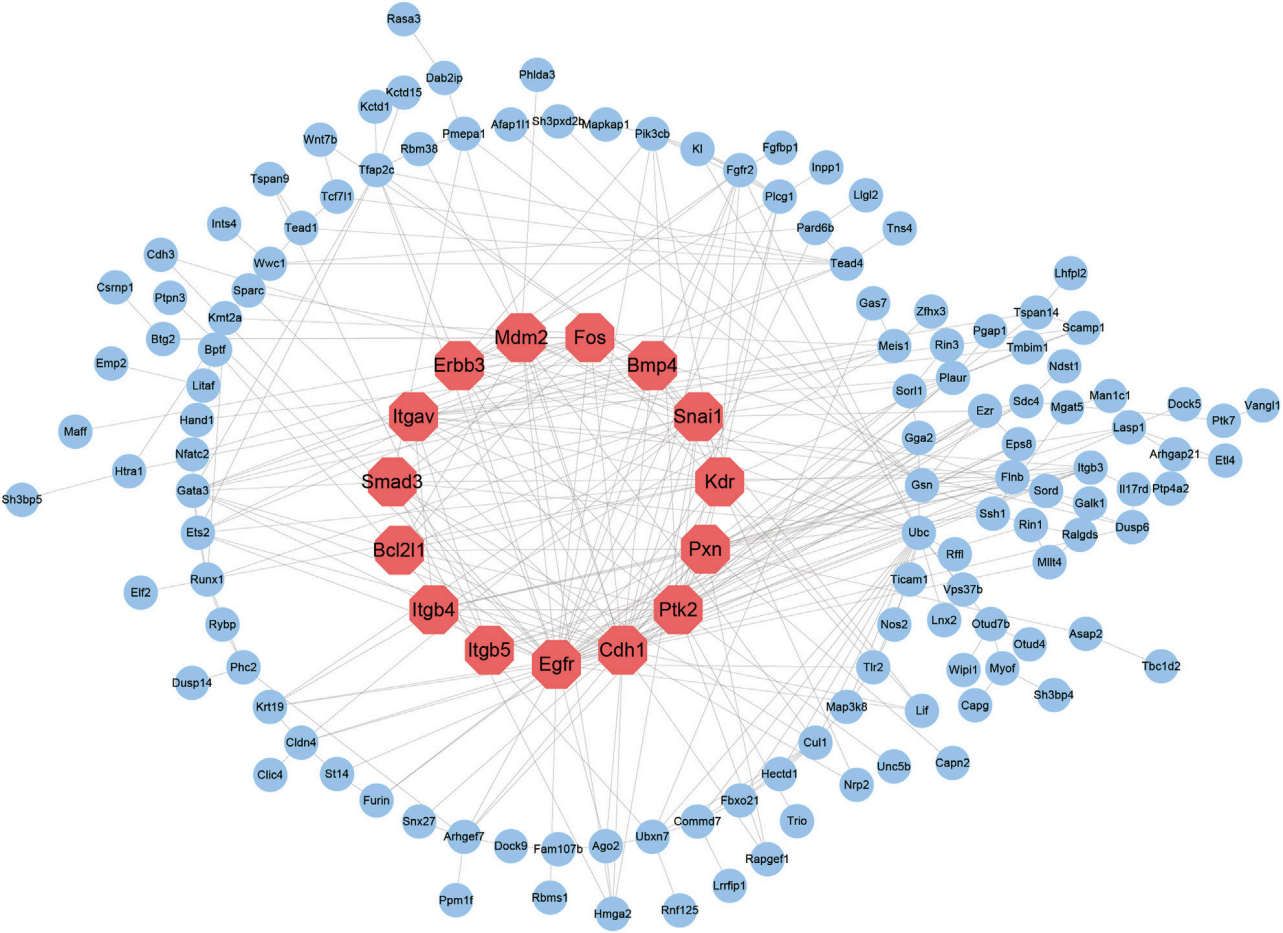
Establishment of the TSC-specific SE PPI network. The top 15 nodes with the highest degrees of connectivity are marked in red, and the node sizes represent the degrees.

Cell differentiation is a process of coordinated polygenic regulation, and intergenic interactions play a significant role in cell differentiation. The STRING database was used to establish a PPI network that included 228 genes, as mentioned above. A PPI network containing 141 nodes and 333 edges was constructed. We used CytoHubba to assess the proteins in the PPI network to identify key genes. Ranked by the degree of connectivity, the top 15 genes were the Epidermal growth factor receptor (Egfr), Cadherin 1 (Cdh1), Protein tyrosine kinase 2 (Ptk2), Pxn, Kinase insert domain receptor (Kdr), β3-Integrin (Itgb3), Snail homolog 1 (Snai1), Bcl2-like 1 (Bcl2l1), β4-Integrin (Itgb4), Integrin, alpha V (Itgav), Fructo-oligosaccharides (Fos), β5-Integrin (Itgb5), Mouse double minute 2 (Mdm2), V-Erb-B2 Avian Erythroblastic Leukemia Viral Oncogene Homolog 3 (Erbb3) and Bone morphogenetic protein 4 (BMP4) genes. The log2fc values of these 15 genes were 3.5, 4.2, 1.2, 1.2, 5.2, 2.0, 1.8, 1.7, 1.3, 2.9, 1.5, 3.8, 1.7, 3.5, and 1.2, respectively, supporting the notion that key cell identity genes regulated by SEs play a significant role in TSC differentiation (Figure 5).

### Verification of Chromatin Accessibility Among Key Cell Identity Genes

**FIGURE 6:**
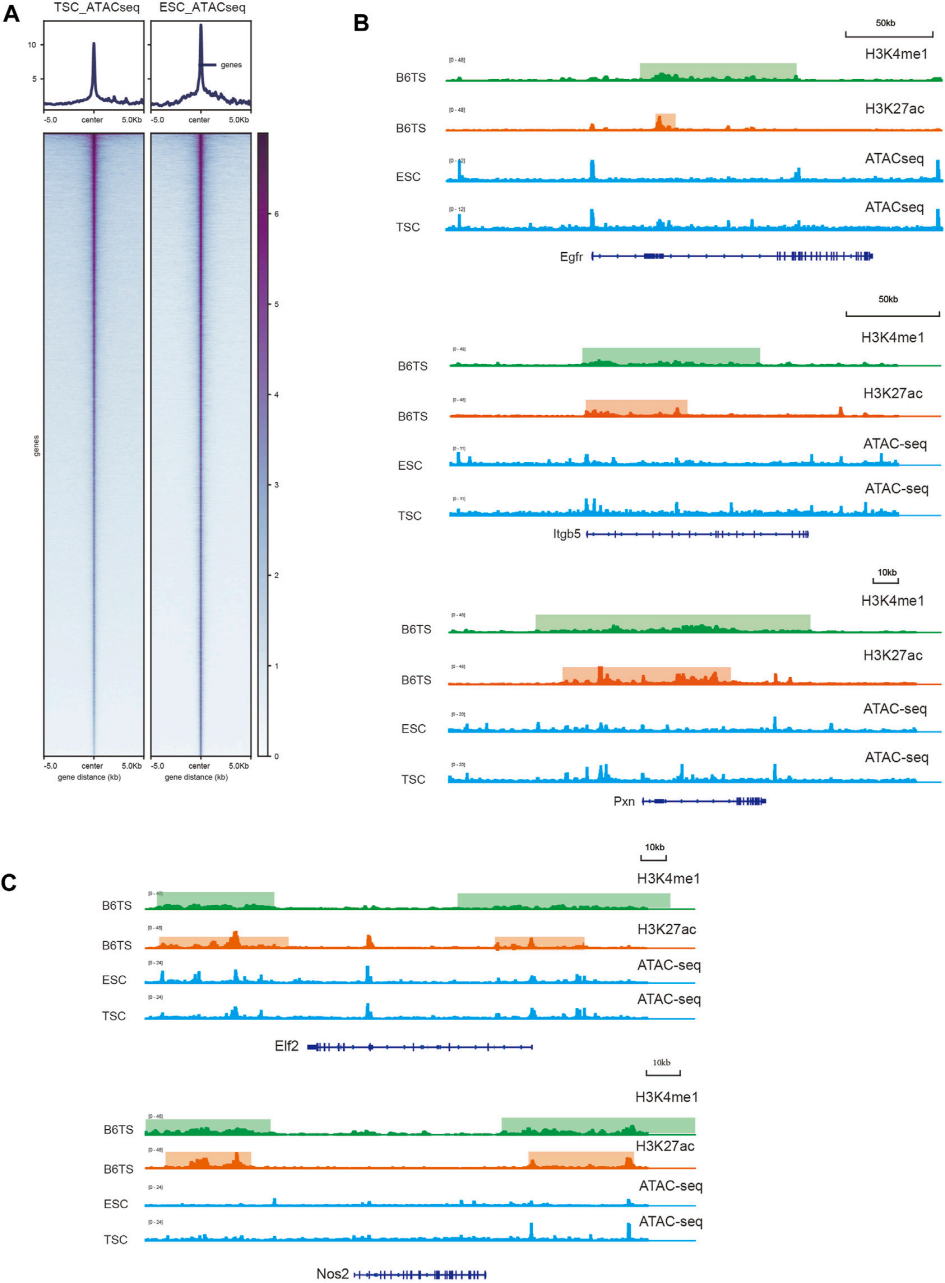
Evaluation of the chromatin accessibility of key cell identity genes. **(A)** Heatmaps showing the general signal levels identified by ATAC-seq in ESCs and TSCs. **(B)** H3K27ac and H3K4me1 signals of TSCs (B6TS) and ATAC-seq signals of ESCs and TSCs at Egfr, Itgb5, Pxn. **(C)** H3K27ac and H3K4me1 signals of TSCs (B6TS) and ATAC-seq signals of ESCs and TSCs at Elf2 and Nos2.

SEs promote gene expression by favoring an open chromatin structure to recruit more transcription factors and cofactors to upregulate genes targeted by SEs. To further clarify whether SEs can regulate key cell identity genes, we integrated ATAC-seq data to visualize the level of chromatin structure accessibility (Figures 6A–C; Supplementary Figures S1A,1B). The ChIP-seq signal graph showed that the locations of TSC-SEs close to key cell identity genes showed stronger H3K27ac and H3K4me1 signals than those observed in ESCs (Figure 6A). Furthermore, the locations of the SEs corresponded to stronger signals of modified histones, which might recruit more transcription factors and cofactors to open the structure of chromatin to facilitate transcription. Thus, we analyzed the results of ATAC-seq in ESCs and TSCs to explore the degree of chromatin opening at the locations of SEs (Figures 6B,C). The integral signal levels of ESCs were higher than those of TSC according to the comparison of ATAC-seq heatmaps. However, among key cell identity genes, the signal levels observed in TSCs were significantly higher than those found in ESCs, indicating higher expression of key cell identity genes owing to the open chromatin structure caused by SEs. To further prove the reliability of the key cell identity genes, we used ChIP-seq and ATAC-seq data from GSE110950, the same dataset as the RNA-seq data, for further verification. Egfr, Itgb5 and Pxn showed SEs enrichment and increased chromatin accessibility (Supplementary Figure S1A), In contrast to no SEs and changes in chromatin accessibility at Elf2 and Nos2 (Supplementary Figure S1B) during TSC differentiation which were in consistent with the previous results.

## Discussion

SEs were identified by analyzing H3K27ac and H3K4me1 ChIP-seq data, and crucial TSC identity genes were discovered through an integrated analysis with RNA-seq data. The SE-associated upregulated genes were subsequently subjected to GO enrichment analysis and KEGG pathway enrichment analysis, further proving that these genes were closely related to the functions of TSCs. A PPI network was constructed using the TSC identity genes, and 15 key cell identity genes, including Egfr, Cdh1, Ptk2, Pxn, Kdr, Itgb3, Snai1, Bcl2l1, Itgb4, Itgav, Fos, Itgb5, Mdm2, Erbb3 and BMP4, were identified among these genes. The chromatin accessibility of these key cell identity genes was assessed by ATAC-seq before and after TSC differentiation, verifying the existence of more open chromatin structures in the SE regions of the key cell identity genes after TSC differentiation and indicating the important roles of key cell identity genes in regulating TSC differentiation.

### Super Enhancers Participate in the Regulation of TSC Differentiation

SEs are occupied by various transcription factors, cofactors, and histone modifications (Whyte et al., 2013; Khan and Zhang, 2019; Deng et al., 2020). Compared with TEs, SEs can not only promote gene transcription more efficiently (Bae et al., 2016) but also regulate cell-type-specific gene regulatory networks (Hnisz et al., 2013), define cell identity and contribute to the development of diseases (Niederriter et al., 2015; Kang et al., 2020) such as neuroblastoma (van Groningen et al., 2017) and cancer. Moreover, studies have shown that SEs play key roles in TSC differentiation (Okae et al., 2018). Therefore, we speculated that SEs are closely connected with the process of TSC differentiation.

SEs were first and most thoroughly identified in ESCs (Hnisz et al., 2013; Whyte et al., 2013), but it remains unknown what roles SEs and TEs play in TSC differentiation. Our study compared the landscapes of SEs and TEs based on ChIP-seq analysis. The mean signal intensity of SE-activated histone modifications was higher than that of TEs in TSCs. The proportion of SEs with short distances to TSSs and promoters was larger than that of TEs, suggesting that SEs play a more important role in genetic transcription than TEs. These results are in accordance with previous studies (Whyte et al., 2013), and we further verified these results in TSC differentiation.

### The Critical Processes and Signaling Pathways in Trophoblast Stem Cells Differentiation

As shown in the KEGG pathway analysis of DEGs, the enriched signaling pathways were those regulating the pluripotency of stem cells, the Hippo signaling pathway and the focal adhesion signaling pathway. Previous studies have evaluated signaling pathways regulating the pluripotency of stem cells, including the LIF/STAT3, Wnt/b-catenin, FGF/ERK, TGF/SMAD and PKC signaling pathways. These three signaling pathways not only maintain ESC self-renewal but are also related to the differentiation of TSCs (Huang et al., 2015). The next most enriched signaling pathways were the Hippo and focal adhesion signaling pathway. The Hippo signaling pathway is a complex signaling network with more than 30 components. The functions of the Hippo pathway have been reported to regulate cell migration, cell differentiation and cell-cell contact, which could contribute to the differentiation of TSCs (Meng et al., 2016; Patel et al., 2017; Ma et al., 2019). Focal adhesion kinase (FAK), a nonreceptor tyrosine kinase with kinase activity and scaffolding function, is involved in proliferation, adhesion, invasion, migration and angiogenesis (Zhang et al., 2021). These processes are also related to cell differentiation (Romani et al., 2021; Zhu et al., 2014). Moreover, some studies have shown that the Hippo component YAP can promote focal adhesion (Shen et al., 2018). This finding indicates that the Hippo pathway may regulate the differentiation of TSCs along with the focal adhesion pathway. According to the results of GO pathway analysis, relevant pathways are involved in the regulation of cell migration, cell adhesion and focal adhesion, which are closely related to the differentiation of TSCs (Romani et al., 2021).

### Super Enhancers Identified the Key Cell Identity Genes of Trophoblast Stem Cells

Based on the intersection of the upregulated genes identified via RNA-seq and SE-targeted genes identified via ChIP-seq, a total of 228 SE-targeted upregulated genes were identified, which we considered TSC identity genes. This demonstrated that the upregulation of these 228 genes at the transcriptional level was due to the increasing levels of SEs during the process of differentiation. Next, we conducted functional enrichment analysis using these TSC identity genes. The KEGG pathway analysis of TSC-SEs revealed that the most enriched signaling pathway that regulating the actin cytoskeleton. The actin cytoskeleton is vital for maintaining the shape and structure of eukaryotic cells, and it contributes to cell migration, cell differentiation, cell interaction and cell division (Rottner et al., 2017; Balta et al., 2020). KEGG pathway analysis also showed enrichment of the focal adhesion signaling pathways and signaling pathways regulating the pluripotency of stem cells. As mentioned above, these two signaling pathways are closely related to the differentiation of TSCs. According to the results of GO pathway analysis, relevant pathways involved in cell adhesion, the positive regulation of cell migration and the positive regulation of cell proliferation were enriched. Combined with the GSEA results, it was inferred that cell adhesion, cell migration and the EGFR signaling pathway were positively associated with the differentiation of TSCs. Hence, these results were similar to those of the functional enrichment analysis of TSC-SEs, illustrating that TSC identity genes were accurately identified. We also screened the key signaling pathways related to TSC differentiation, which were unclear in previous studies. Furthermore, we filtered key genes in the PPI network using CytoHubba, revealing the top 15 significantly upregulated genes ant their log2fc values.

Epidermal growth factor receptor (EGFR) belongs to the ERBB family of tyrosine kinase receptors, and the EGFR signaling pathway is activated by epidermal growth factor (EGF). Previous studies have shown that EGFR regulates focal adhesion, the actin cytoskeleton and cell differentiation(Roskoski, 2019; Rao et al., 2020; Wu and Zhang, 2020). However, it is still unknown what roles EGFR plays in TSC differentiation. According to our results, EGFR is a key cell identity gene, and the focal adhesion and regulation of the actin cytoskeleton signaling pathways are the key signaling pathways involved in TSC differentiation.

The ITGB5 gene is a member of the integrin β (ITGB) superfamily (Zhuang et al., 2020) and encodes integrin β5, which is closely related to cell adhesion (Wang et al., 2019). A previous study showed that ITGB5 contributes to the migration and invasion of glioma cells in tube formation by endothelial cells (Zhang et al., 2019). Therefore, we speculated that ITGB5 plays a crucial role in TSC differentiation by positively regulating cell adhesion, migration and invasion.

PXN is a focal adhesion adapter protein that is involved in many cell functions, such as focal adhesion and migration, and regulates the organization of the actin cytoskeleton (Webb et al., 2004; López-Colomé et al., 2017; Legerstee et al., 2019). Our results also proved that PXN occupies a central position in the PPI network; furthermore, it is closely related to the primary enriched signaling pathways mentioned above. Thus, we believe that PXN can regulate TSC differentiation by regulating focal adhesion, cell migration and actin cytoskeleton organization.

### The Chromatin Accessibility of Key Cell Identity Genes Increased During TSC Differentiation

The more open chromatin structures in the SE regions are one of the intrinsic mechanisms promoting transcription (Michida et al., 2020) and favoring the recruitment of more transcription factors and cofactors (Thomson et al., 1998). To determine whether SEs can regulate key cell identity genes, we integrated ATAC-seq data to reveal the degree of chromatin accessibility. The ChIP-seq signal graph showed that SEs were present in key cell identity genes such as the EGFR, ITGB5 and PXN genes. This further demonstrated that the SEs at these positions could recruit many transcription factors and cofactors to change and open the chromatin structure to promote transcription. The heatmap showed the ATAC-seq signals between TSCs and ESCs, and the integrated signal of ESCs was stronger than that of TSCs. However, the ATAC-seq signals of ESCs were weaker than those of TSCs when read in association with key cell identity genes. Therefore, the increasing ATAC-seq signals of key cell identity genes were due to the dynamic transformation of SEs and not to the increase in the integrated ATAC-seq signal. This further demonstrates that SEs upregulate the expression of key cell identity genes. Chromatin opening at key cell identity genes regulated by SEs is the mechanism enhancing gene expression. These results are in accordance with previous studies and further contribute to the understanding of TSC differentiation.

## Data Availability

The original contributions presented in the study are included in the article/Supplementary Material, further inquiries can be directed to the corresponding author.

## Supplementary Material

Supplementary Figure S1 Evaluation of the chromatin accessibility of key cell identity genes. (A) H3K27ac and H3K4me1 signals of TSCs and ATAC-seq signals of ESCs and TSCs from GSE110950 at Egfr, Itgb5, Pxn. (B) H3K27ac and H3K4me1 signals of TSCs and ATAC-seq signals of ESCs and TSCs from GSE110950 at Elf2 and Nos2.

## Glossary

ATAC-seq
ATAC-sequencing
Bcl2l1: Bcl2-like 1
BMP4: Bone morphogenetic protein
Cdh1: Cadherin 1
ChIP-seq: Chromatin immunoprecipitation sequencing
CytT: mononuclear cytotrophoblasts
DEGs: differentially expressed genes
Egfr: Epidermal growth factor receptor
EGFR: Epidermal growth factor receptor
ESC: Embryonic stem cell
Erbb3: V-Erb-B2 Avian Erythroblastic Leukemia Viral Oncogene Homolog 3
Fos: Fructo-oligosaccharides
GEO: Gene Expression Ominbus
GO: Gene ontology
GO_CC: GO cellular component category
GO_BP: GO biological process category
GO_MF: GO molecular function category
H3K27ac: Histone 3 lysine 27 acetylation
H3K4me1: Monomethylation of lysine 4 of histone H3
ICM: inner cell mass
Itgav: Integrin, alpha V
Itgb3: β3-Integrin
Snai1: Snail homolog 1
Itgb4: β4-Integrin
Itgb5: β5-Integrin
ITGB5: Integrin β5
Kdr: Kinase insert domain receptor
KEGG: Kyoto Encyclopedia of Genes and Genomes
Mdm2: Mouse double minute 2
PPI: protein-protein interaction
Ptk2: Protein tyrosine kinase 2
Pxn: Paxilllin
RNA-seq: RNA sequencing
SEs: Super enhancers; TEs: typical enhancers
SynT: multinuclear syncytiotrophoblast
TSC: Trophoblast stem cell
TSS: transcription start sites
