# Supplementary figures and images for: Super Enhancer Profiles Identify Key Cell Identity Genes During Differentiation From Embryonic Stem Cells to Trophoblast Stem Cells Super Enhencers in Trophoblast Differentiation
Source: Front Genet. 2021 Oct 12;12:762529. doi: 10.3389/fgene.2021.762529 (PMC8546299; doi:10.3389/fgene.2021.762529)

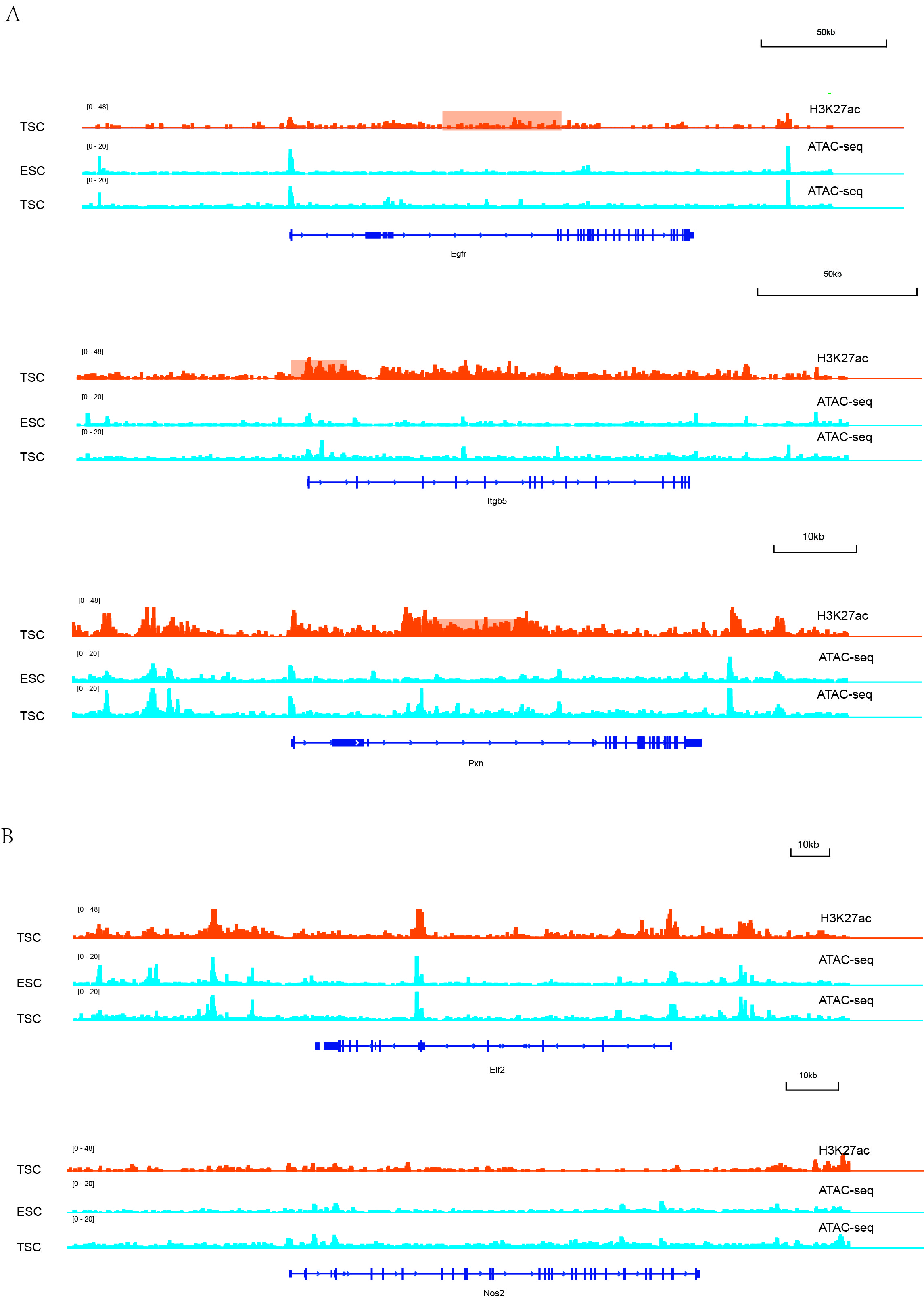

Supplement: Supplementary file 1 [file Image1.tiff]
